# Prognostic Differences in Patients with Solitary and Multiple Spinal Metastases

**DOI:** 10.1111/os.12470

**Published:** 2019-06-09

**Authors:** Deng‐xing Lun, Li‐na Xu, Feng Wang, Xiong‐gang Yang, Xiu‐chun Yu, Guo‐chuan Zhang, Yong‐cheng Hu

**Affiliations:** ^1^ Department of Spine Surgery Weifang People's Hospital Weifang China; ^2^ Department of Respiratory Medicine Weifang People's Hospital Weifang China; ^3^ Graduate School Tianjin Medical University Tianjin China; ^4^ Department of Orthopaedic Oncology Jinan Military General Hospital Jinan China; ^5^ Department of Orthopaedic Surgery The Third Hospital of Hebei Medical University Shijiazhuang China; ^6^ Department of Bone Oncology Tianjin Hospital Tianjin China

**Keywords:** Karnofsky performance status, Metastatic spinal cord compression, Multiple spinal metastasis, Prognostic factors, Spinal metastasis

## Abstract

**Objectives:**

To investigate the association between the number of metastases to the spine and survival in patients with metastatic spinal cord compression (MSCC), as well as the prognosis difference between patients with solitary spinal metastasis (SSM) and multiple spinal metastases (MSM).

**Methods:**

Three institutional databases were searched to identify all patients who had undergone spinal surgery for metastatic spinal tumors between March 2002 and June 2010. As well as age and gender, preoperative medical conditions were collected from medical records, including primary tumor, preoperative Frankel score, other bone metastases, preoperative Karnofsky performance status (KPS), number of involved vertebrae, pathological fracture metastasis site, serum albumin, sphincter dysfunction and the time of developing motor deficits before surgery. Survival data were obtained from medical records or *via* telephone follow‐ups. Univariate and multivariate predictors of overall survival for each group were assessed using the Cox proportional hazards model.

**Results:**

The median postoperative survival time was 6.0 ± 0.6 months (95% confidence interval [CI] 4.8–7.2) in patients with SSM and 7.0 ± 1.0 months (95% CI 5.1–8.9) in patients with MSM (*P* = 0.238). The difference in survival was not significant between groups. Furthermore, univariate analysis showed that the number of spinal metastases had no significant association with survival (*P* = 0.075). Primary tumor (*P* = 0.004) and preoperative KPS (*P* < 0.001) were independent prognostic factors in the whole cohort; primary tumor (*P* = 0.020), time of developing motor deficit (*P* = 0.041) and preoperative KPS (*P* = 0.038) were independent prognostic factors in patients with SSM; while preoperative KPS (*P* = 0.001) and serum album level (*P* < 0.001) were independent prognostic factors in patients with MSM.

**Conclusion:**

The number of spinal metastases has not proven to be useful in predicting the prognosis for patients with MSCC. Consequently, more aggressive operations should be considered for patients with multiple spinal metastases.

## Introduction

Taking into consideration advanced treatment technologies and extended survival time, the aim of surgical treatment for patients with metastatic spinal cord compression (MSCC) should no longer be only about relieving pain and restoring function but also about achieving better local control and reducing reoperation rates over the remaining lifetime. Total en bloc spondylectomy (TES) has been widely accepted as the optimal treatment with reference to control of local recurrence rate because of complete resection of spinal tumors^1^, ^2^, ^3^, ^4^. However, TES is indicated as the primary treatment method only for solitary spinal metastasis, with a life expectancy ≥6 months^5^, ^6^, ^7^, while the first‐choice treatment for multiple spinal metastases is still palliative surgery no matter how long the life of patients has been extended^8^, ^9^.

Apart from technical challenges, the main reason why patients with multiple spinal metastases are not considered for TES is shorter survival times^9^. Generally, it is accepted that life expectancy drives treatment regimens for spine metastasis^10^. Multiple spinal metastases, a symbol of more aggressive tumors, are usually regarded as advanced stage cancer. Thus, patients with multiple spinal metastases tend to have shorter survival rates that often make them unsuitable for radical surgery. Nevertheless, there is still considerable controversy regarding the association between the number of metastases to the spine and survival prognosis^8^, ^9^, ^10^, ^11^, ^12^, ^13^, ^14^, ^15^, ^16^. For example, significant differences have been observed among several commonly used scoring systems^8^, ^9^, ^11^, ^12^, ^13^, ^14^, ^15^, ^16^. Tomita *et al*.^8^, Tokuhashi *et al*.^9^, ^11^ and Sioutos *et al*.^12^ reported that patients with multiple spinal metastases had a poor prognosis compared to those with a solitary spinal lesion. They also considered the number of metastatic tumors as one of the prognostic factors in their scoring systems; however, Bauer^13^, modified Bauer^14^, Van der Linden *et al*.^15^ and Rades *et al*.^16^ have omitted the number of spinal metastases from their scoring systems.

Given the aforementioned conflicting results, the goal of the present study is: (i) to further identify the quantitative role of spinal metastases in predicting survival rates for patients with spinal metastases; and (ii) to investigate differences in prognosis between patients with solitary and multiple spinal metastases.

## Methods

This study was approved by the authors’ hospital ethics committee. All dates were reviewed from three different research centers between March 2002 and June 2010.

### *Inclusion Criteria and Exclusion Criteria*

Inclusion criteria followed the PICOS principle: (i) Participant: patients with spinal metastasis, including patients with medically intractable pain, rapidly progressive neurological deterioration, or evidence of clinical or radiographic instability; (ii) Intervention: open surgery with or without internal fixation; (iii) Comparison: prognostic factors was compared between the SSM group and the MSM group; (iv) Outcome: survival rate and prognostic factors; and (vi) Study design: retrospective study.

Exclusion criteria: (i) patients with spinal metastasis without cord compression; (ii) those who received radiotherapy or were subjected to revision procedures; (iii) patients having vertebroplasty or kyphoplasty; (iv) patients with life expectancies <3 month and patients with poor medical condition who would not be able to survive the operation.

### *Imaging Examination*

Magnetic resonance imaging (MRI) was used to investigate the number of spinal metastases. Other staging studies included computed tomography (CT) of the chest, the abdomen, and the pelvis. In addition, radionuclide bone scans were used to identify whether metastasis was present in other parts of the body. Patients with single or two observed continuous spinal metastases (regardless of whether metastases in other parts of the body were diagnosed) were classified as having solitary spinal metastasis (SSM), while those with non‐consecutive spinal metastases and ≥ 3 consecutive spinal metastases were classified as having multiple spinal metastases (MSM).

### *Prognostic Factors*

Survival data were obtained based on medical records or telephone follow‐up, or from governmental cancer registry systems. These patients were divided into two groups according to the number of spinal metastases. Demographic data and preoperative medical conditions were collected from medical records or *via* telephone follow‐ups. Several prognostic factors were analyzed, and each variable was categorized into two or three subgroups including age (<65 *vs* ≥65 years), gender (female *vs* male), primary tumor (rapid *vs* moderate *vs* slow), preoperative Frankel score (A‐C *vs* D‐E), other bone metastases (no *vs* yes), preoperative KPS (10–40 *vs* 50–70 *vs* 80–100), number of involved vertebrae (solitary *vs* multiple), pathological fracture (no *vs* yes), metastasis site (cervical *vs* cervical), serum albumin (<35 *vs* ≥35 g/L), sphincter dysfunction (no *vs* yes), and the time of developing motor deficits before surgery (≤5 *vs* >5 days). Based on Tomita *et al*.^8^, primary cancer types were categorized by tumor growth: slow (breast, prostate, and thyroid), moderate (kidney and uterus), and rapid (lung, colon, liver, gastric cancer, or other cancers).

### *Outcome Measures*

The postoperative survival was defined as the time between the date of surgery and the patient's death or the latest follow‐up.

Preoperative neurological function was graded based on Frankel grade (patients with Frankel D and E were able to walk). Time of developing motor deficits was defined as the time between deterioration of motor function to surgery. Deterioration of motor function was defined as a change of at least one Frankel grade.

### *Statistical Analysis*

Mean values were reported as mean ± standard deviation. Median values were reported with range. The characteristic of the two groups were compared using the *χ* ^2^‐test. The Kaplan–Meier method was used to estimate postoperative survival and survival time. Univariate and multivariate predictors of overall survival for each group were assessed using the Cox proportional hazards model. Variables significant at *P* < 0.01 in the univariate analysis were tested through a backward stepwise selection process for their independent effect on overall survival. Rate ratios and their 95% confidence intervals (CI) were computed. Odds ratios and their 95% CI were computed. *P*‐value <0.05 was considered significantly different.

## Results

### *Patient Characteristics*

The characteristics related to both groups are summarized in Table 1. There were 102 men and 67 women, and the mean age of patients was 59.6 ± 10.5 years (range, 29–81 years). At the time of spinal surgery, 78 patients had SSM, while 91 patients had MSM. The primary cancers were lung cancer (73 patients, 43%), breast cancer (13 patients, 8%), renal cancer (12 patients, 7%), hepatic cancer (10 patients, 6%), gastrointestinal cancer (9 patients, 5%), prostate cancer (7 patients, 4%), unidentified primary tumor (27 patients, 16%), and others (18 patients, 11%, including 5 esophageal, 4 thyroid and 1 each of nasopharyngeal, cervical carcinoma, urinary bladder, ureter, parotid, sublingual gland, epinephros, and thymus gland cancer).

**Table 1:** Baseline characteristics of the study population

Variables	All patients	Patients with solitary vertebrae metastasis	Patients with multiple vertebrae metastasis	*P‐*value
Number	169	78	91	—
Age (mean ± SD)	59.6 ± 10.5	60.1 ± 11.1	59.2 ± 10.0	0.560
Age‐N (%)				0.921
<65	109	50	59	
≥65	60	28	32	
Gender‐N (%)				0.061
Male	102	83	55	
Female	67	31	36	
Systematic co‐morbidity‐N (%)				0.781
Yes	56	25	31	
No	113	53	60	
Primary tumor‐N (%)				0.192
Group A (rapid)	78	40	38	
Group B (moderate)	65	30	35	
Group C (slow)	26	8	18	
Location of involved vertebrae‐N (%)				0.597
Cervical	22	9	13	
Non‐cervical	147	69	78	
Preoperative Frankel grade‐N (%)				0.128
A–C	37	13	24	
D–E	132	65	67	
Extrospinal bone metastasis‐N (%)				0.055
Yes	113	58	55	
No	56	20	36	
Pathological fracture‐N (%)				0.632
Yes	33	14	19	
No	136	64	72	
Visceral metastasis‐N (%)				0.698
Yes	41	20	21	
No	128	58	70	
Preoperative KPS‐N (%)				0.564
10–40	19	10	9	
50–70	94	40	54	
80–100	56	28	28	
Time developing motor deficit‐N (%)				0.035*
≤5 days	121	62	59	
>5 days	48	16	32	
Urinary retention/incontinence‐N (%)				0.563
Yes	13	7	6	
No	156	71	85	
Serum album level (g/L)‐N (%)				0.111
<35 g/L	17	11	6	
≥35 g/L	96	42	54	
Adjuvant therapy‐N (%)				0.127
Yes	125	64	61	
No	44	19	25	

* Statistical significance; N, number.

### *Survival Rate*

**Figure 1:**
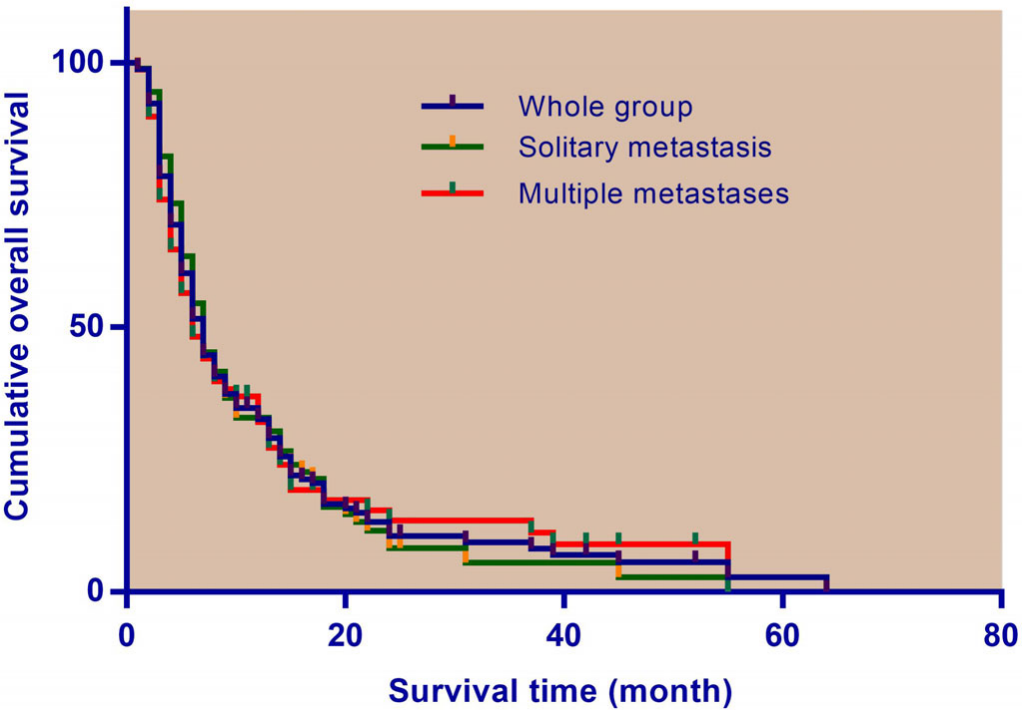
Kaplan–Meier survival analysis for whole group, and groups with solitary and multiple spinal metastases (showing no significant difference in survival between groups).

Overall median survival was 6.0 ± 0.6 (95% CI 4.8–7.2) months in patients with SSM, while it was 7.0 ± 1.0 (95% CI 5.1–8.9) months in patients with MSM. The difference in survival between groups was not significant (HR 1.23, 95% CI 0.87–1.74, *P* = 0.238). Figure 1 shows the Kaplan–Meier survival estimates categorized by number of spinal metastases. The overall 6 and 12‐month survival rates were, respectively: 51.6% and 32.7% in all patients; 54.8% and 34.8% in patients with SSM; and 56.8% and 38.5% in patients with MSM.

### *Comparison of Prognostic Factors between Two Groups*

The patients with multiple metastases had significantly shorter time developing motor deficit: 62 (79.5%) *vs* 59 (64.8%) patients ≤5 days, *P* < 0.035 compared to those with SSM. No statistically significant difference between the two groups was observed in any of the following characteristics: primary tumor (*P* = 0.192), age (*P* = 0.921), gender (*P* = 0.061), preoperative Frankel score (*P* = 0.564), other bone metastases (*P* = 0.055), postoperative KPS (*P* = 0.384), number of involved vertebrae (*P* = 0.826), pathological fracture (*P* = 0.632), visceral metastasis (*P* = 0.698), sphincter dysfunction (*P* = 0.563), and serum album level (*P* = 0.111).

### *Overall Prognostic Factors*

Univariate analyses identified significant prognostic factors for OS, which included primary tumor (*P* = 0.015), preoperative KPS (*P* < 0.001) and Frankel grade (*P* < 0.001), location of involved vertebrae (*P* = 0.021), and adjuvant radiotherapy (*P* = 0.001) (Table 2). Nevertheless, multivariable analysis with maximal model found that prognostic factors for OS included primary tumor (*P* = 0.004) and preoperative KPS (*P* < 0.001) (Table 3).

**Table 2:** Results of univariate analysis by log‐rank test

Variables	Whole cohort (%)		Median (m)	*P*‐value	Patients with solitary vertebrae metastasis (%)		Median (m)	*P*‐value	Patients with multiple vertebrae metastasis (%)		Median (m)	*P*‐value
	6 m	12 m			6 m	12 m			6 m	12 m		
Primary tumor												
Group A (rapid)	41.1	26.5	6.0 ± 0.5	0.015*	35.9	19.2	6.0 ± 0.5	0.076	47.2	35.0	6.0 ± 1.1	0.264
Group B (moderate)	54.9	32.6	7.0 ± 1.0		56.7	39.0	7.0 ± 1.9		53.5	33.5	7.0 ± 1.4	
Group C (slow)	73.1	50.0	10.0 ± 3.5		87.5	62.5	15.0 ± 4.2		66.6	44.4	9.0 ± 1.1	
Gender												
Male	59.8	36.3	6.0 ± 0.7	0.547	43.6	25.0	6.0 ± 0.7	0.568	48.6	36.7	6.0 ± 1.0	0.748
Female	46.2	30.3	8.0 ± 1.1		58.1	33.9	7.0 ± 1.1		61.4	38.5	8.0 ± 1.8	
Age												
≥65	48.6	32.6	6.0 ± 1.1	0.931	44.6	33.1	7.0 ± 1.4	0.914	52.0	32.2	5 ± 1.7	0.993
<65	53.2	32.6	7.0 ± 0.6		52.0	25.7	7.0 ± 1.3		54.6	38.7	7 ± 0.6	
Systematic comorbidity												
Yes	46.4	32.9	6.0 ± 0.9	0.721	36.0	31.5	5.0 ± 0.6		54.8	33.9	7.0 ± 1.2	
No	54.3	32.6	7.0 ± 0.7		55.8	27.1	7.0 ± 0.6	0.704	52.9	37.6	7.0 ± 1.6	0.803
Visceral metastasis												
Yes	39.0	23.3	5.0 ± 0.5	0.080	40.0	24.0	5.0 ± 0.5	0.284	38.1	22.9	5.0 ± 0.7	0.155
No	55.7	35.8	7.0 ± 0.8		52.6	30.3	7.0 ± 0.7		58.4	40.5	8.0 ± 1.7	
Preoperative Frankel grade												
A‐C	42.4	13.8	5.0 ± 1.3	<0.001*	38.5	0.0	5.0 ± 1.2	0.019*	44.6	17.8	4.0 ± 1.7	0.007*
D‐E	54.2	37.6	7.0 ± 0.8		51.6	33.3	7.0 ± 0.6		56.8	41.7	8.0 ± 2.5	
Preoperative KPS												
80–100	58.3	43.3	8.0 ± 2.1	<0.001*	59.3	43.7	10.0 ± 2.4	0.004*	55.6	40.8	8.0 ± 1.7	<0.001*
50–70	53.9	32.0	7.0 ± 0.7		50.0	23.4	6.0 ± 0.6		57.8	40.2	8.0 ± 2.4	
10–40	21.1	0.0	3.0 ± 0.4		20.0	10.0	3.0 ± 0.8		22.2	0.0	3.0 ± 0.7	
Extrospinal bone metastasis												
Yes	49.0	34.2	6.0 ± 0.8	0.234	50.0	27.8	6.0 ± 0.8	0.492	48.6	36.8	6.0 ± 0.9	0.096
No	53.7	31.5	7.0 ± 0.7		49.1	29.2	6.0 ± 0.8		61.8	34.9	8.0 ± 1.4	
Pathological fracture												
Yes	61.4	39.9	9.0 ± 2.1	0.297	61.5	27.7	8.0 ± 1.7	0.659	61.3	48.3	12.0 ± 4.1	0.367
No	49.3	31.0	6.0 ± 0.5		46.9	28.5	6.0 ± 0.7		51.6	33.2	7.0 ± 0.9	
Number of involved vertebrae												
Solitary	54.8	34.8	6.0 ± 0.6	0.075	—	—	—	—	—	—	—	—
Multiple	56.8	38.5	7.0 ± 1.0		—	—	—	—	—	—	—	—
Time developing motor deficit												
≤5 days	48.2	33.1	6.0 ± 0.5	0.370	51.6	34.7	7.0 ± 0.9	0.039*	44.4	31.2	6.0 ± 0.6	0.078
>5 days	60.8	31.4	8.0 ± 1.0		40.0	0.0	5.0 ± 1.3		71.5	46.2	12.0 ± 2.5	
Urinary retention/incontinence												
Yes	38.5	15.4	4.0 ± 0.9	0.131	57.1	14.3	7.0 ± 2.6	0.428	16.7	16.7	3.0 ± 0.6	0.188
No	52.7	34.2	7.0 ± 0.6		48.6	30.1	6.0 ± 0.6		56.2	37.6	7.0 ± 1.3	
Serum album level												
≥35g/L	55.2	33.5	7.0 ± 0.9	0.300	52.4	24.1	7.0 ± 0.8	0.491	58.3	42.0	8.0 ± 2.6	0.001*
<35g/L	44.4	27.8	4.0 ± 1.0		54.5	36.4	7.0 ± 1.6		16.7	0.0	3.0 ± 0.4	
Location of involved vertebrae												
Cervical	66.8	50.9	14.0 ± 7.0	0.021*	55.6	41.7	10.0 ± 5.0	0.118	75.5	55.6	14.0 ± 11.2	0.120
Non‐cervical	49.3	39.1	6.0 ± 0.6		48.6	26.8	6.0 ± 0.7		50.1	32.9	7.0 ± 1.1	

* Statistical significance.

KPS, Karnofsky performance status; m, months; −, not reported.

**Table 3:** Significant prognostic factors in multivariate analysis by COX hazard proportional model in the whole cohort

Prognostic factors	Hazard ratio	95% confidence interval	*P*‐value
Primary tumor			
Group C (slow)	1		0.004*
Group B (moderate)	1.72	1.03–2.87	0.037*
Group A (rapid)	2.32	1.40–3.83	0.001*
Preoperative KPS			
80–100	1		<0.001*
50–70	1.25	0.86–1.82	0.234
10–40	4.95	2.66–9.22	<0.001*

KPS, Karnofsky performance status.

* Statistical significance.

### *Prognostic Factors for Patients with Solitary Spinal Metastasis*

Preoperative Frankel score (*P* = 0.019), preoperative KPS (*P* = 0.004), time of developing motor deficit (*P* = 0.039), and adjuvant therapy (*P* = 0.045) were potential prognostic factors, as shown by univariate analysis (Table 2). Furthermore, the multivariate Cox regression model showed that primary tumor (*P* = 0.020), time of developing motor deficit (*P* = 0.041), and preoperative KPS (*P* = 0.038) were significant prognostic factors for MSCC (Table 4).

**Table 4:** Significant prognostic factors on multivariate analysis in patients with solitary spinal metastasis

Prognostic factors	Hazard ratio	95% confidence interval	*P*‐value
Primary tumor			
Group C (slow)	1		0.020*
Group B (moderate)	1.85	0.75–4.55	0.182
Group A (rapid)	3.01	1.28–7.09	0.012*
Time developing motor deficit			
≤5 days	1		
>5 days	2.07	1.03–4.17	0.041*
Preoperative KPS			
80–100	1		0.038*
50–70	1.65	0.94–2.88	0.081
10–40	3.02	1.25–7.32	0.014*

* Statistical significance.

KPS, Karnofsky performance status.

### *Prognostic Factors for Patients with Multiple Spinal Metastases*

Univariate analysis suggested that potential prognostic factors were the following: preoperative Frankel score (*P* = 0.007) and KPS (*P* < 0.001), serum album level (*P* = 0.001) and adjuvant therapy (*P* < 0.001) (Table 2). In addition, the multivariate Cox regression model showed that preoperative KPS (*P* = 0.001) and serum album level (*P* < 0.001) were significantly correlated with survival time (Table 5).

**Table 5:** Significant prognostic factors on multivariate analysis in patients with multiple spinal metastases

Prognostic factors	Hazard ratio	95% confidence interval	*P*‐value
Preoperative KPS			
80–100	1		0.001*
50–70	2.00	1.02–3.91	0.042*
10–40	2.90	1.17–7.23	0.022*
Serum album level			
≥35 g/L	1		
<35 g/L	6.90	2.49–19.14	<0.001*

* Statistical significance.

KPS, Karnofsky performance status.

## Discussion

With refinement in surgical techniques, improvement in systemic therapies, and optimization of scoring systems used to determine optimal treatments, the survival time of patients with MSCC has gradually increased^17^, which, in turn, makes the current scoring systems ineffective and obsolete; thus, this event is constantly repeated^14^. Therefore, re‐predicting survival or re‐identifying new risk factors based on current medical standards are strategic approaches for selecting emerging treatment modalities. In the present study, we re‐identified the quantitative role of spinal metastases in predicting overall survival in patients with spinal metastases. To the best of our knowledge, this is the first study to investigate prognostic differences between patients with solitary and multiple spinal metastases.

### *Effect of Number of Metastases on Survival*

The presence of MSM often implies that the underlying disease might be incurable given it is usually a part of already spread cancer disease; thus, patients with multiple spinal metastases tend to have shorter survival. The number of affected spinal levels is among six factors that constitute the Tokuhashi prognostic score^9^, ^11^. Chong *et al*. reported that the median survival of patients with ≤3 levels of spinal metastases is 16.0 months; which is significantly longer compared to patients with ≥3 levels of spinal metastases (median of 4.0 months). In addition, Chong and his team identified the quantity of metastases as the only preoperative factor for survival^18^. Furthermore, Aoude *et al*. reviewed 126 patients afflicted with spinal metastases and ranked the importance of six parameters of modified Tokuhashi score by calculating beta weights of regression equation. They found that the number of spinal metastases and visceral metastasis ranked the highest. Other factors, such as general condition, extraspinal metastases, palsy, and primary site, were not found to be associated with survival time^19^. Moreover, in a prospective multicenter cohort study of 922 patients with spinal metastases who underwent surgery, Choi *et al*. found that the number of spinal metastases was strongly associated with patients’ survival, followed by primary tumor type and visceral metastases^20^. Mosele *et al*. predicted postoperative survival factors among 63 patients suffering from renal cancer with MSCC and found that patients with SSM had significantly better survival compared to patients with MSM^21^. Lei *et al*. analyzed postoperative survival factors among 64 patients suffering from lung cancer with MSCC and found that the survival time of patients with 1–2 spinal metastases is 2.46 times longer compared to patients with 3 or more spinal metastases^22^.

Our results revealed that patients with multiple spinal metastases did not have a significantly shorter survival time compared to patients with solitary spinal metastasis, which is completely opposite from certain existing literature and scoring systems 8, 9 and 11. The obtained findings are in accordance with previous reports from the literature^23^, ^24^, ^25^: Yang *et al*. found that the median survival for patients with SSM was 7 months, while it was 5 months for patients with MSM. Multivariate and univariate analyses have both showed that there is no significant difference between the two groups^23^. Zaw *et al*. identified primary tumor and preoperative Eastern Cooperative Oncology Group performance status as the only significant predictors of overall survival, while number of spinal metastasis in connection with survival, in a variety of primary tumors with spinal metastases following spinal surgery, had no statistical significance^24^. Wibmer *et al*. analyzed the predictive value of seven scoring systems, as well as the parameters included in these systems, and found that the number of spinal metastases in the Tokuhashi scoring system did not affect survival rates, regardless of the one versus two spinal metastases, or single versus multiple spinal metastases^25^.

In addition, the solitary or multiple spinal metastases statuses have been demonstrated to have a similar impact on prognosis in different primary tumor types. Rades *et al*. show that quantity of spinal metastases has no significant association with survival of elderly prostate cancer patients with MSCC. Moreover, they found that the 6‐month and 12‐month survival rates for patients with 1–2 spinal metastases, 3–4 spinal metastases, and ≥5 spinal metastases were 65% and 49%, 58% and 49%, and 49% and 39%, respectively^26^. Sellin *et al*. identified postoperative factors that influenced overall survival in 43 patients with thyroid cancer and spinal metastasis, and found that the number of involved spinal levels and visceral metastasis did not significantly affect overall survival^27^. By performing a retrospective review of 21 patients who underwent surgical treatment, Bakker *et al*. found that MSM are not significantly associated with survival in patients with renal cell carcinoma^28^. Moreover, Rades *et al*. conducted a multicenter study based on 356 patients suffering from non‐small cell lung cancer with MSCC and found that the median survival time was 4 months for patients with 1–2 spinal metastases and 3 months for patients with ≥3 spinal metastases^29^. Daniel *et al*. found that multiplicity of spinal lesions and presence of visceral metastases does not affect prognosis for patients with breast cancer, while cervical metastasis is the only independent risk factor^30^. Therefore, the results obtained in the present study are in line with several existing studies arguing that the quantity of spinal metastases does not influence the overall survival of patients with different tumor subtypes.

### *Prognostic Factors of Solitary and Multiple Spinal Metastases*

The current study did not show an association between the number of spinal metastases and decreased survival. The exact cause of the obtained results remains unclear. One possible reason is that advanced treatment strategies, such as targeted therapy, hormonal therapy, chemotherapy, and stereotactic body radiotherapy, can effectively control systemic metastasis and can significantly prolong the survival time for patients with MSCC. Another possible explanation is bias in selection of patients for surgery because higher Tokuhashi scores might have included more patients with a number of spinal metastases that were excluded from surgical consideration. Another possibility is that the number of spinal metastases may not affect the prognosis of one of these primary tumors, which account for a larger proportion in the present study.

Although the number of spinal metastases has not shown the ability to predict the prognosis of patients with MSCC, we did find different prognostic factors in patients with solitary and multiple spinal metastases. For example, the factors influencing survival of patients with solitary spinal metastasis were primary tumor, time of developing motor deficit, and preoperative KPS; in contrast, preoperative KPS and serum album level were significantly associated with survival in patients with multiple spinal metastases. From these findings, it is obvious that besides overall survival in the whole cohort, preoperative KPS affects the survival of patients with single or multiple metastases. This is in line with previous studies that have identified performance status as one of the strongest prognostic factors for survival^31^, ^32^, ^33^. In our study, KPS scores ranging from 80 to 100 were associated with a better prognosis compared to scores ranging from 10 to 40 (HR 4.95, 95% CI 2.66–9.22, *P* < 0.001). Nevertheless, there was a small difference between high and medium score groups (HR 1.25, 95% CI 0.86–1.82, *P* = 0.234). In addition, these same results were found in patients with solitary spinal metastasis. However, in patients with multiple spinal metastases, there were significant survival differences with reference to 80–100 versus 10–40 groups (HR 2.00, 95% CI 1.02–3.91, *P* = 0.042) or 80–100 versus 50–70 groups (HR2.90, 95% CI 1.17–7.23, *P* = 0.022). The obtained results may be related to arbitrarily set KPS group scores. However, further group analysis was not performed because the present study only focused on the association between the number of spinal metastases and survival.

Primary tumor was also identified as a significant prognostic factor for the whole study group and for the group with SSM. This was in line with most of the existing scoring systems that include primary cancers^8^, ^9^, ^11^, and several studies have identified primary cancer diagnosis as the most important prognostic factor in relation to survival^34^, ^35^. Arrigo *et al*. reported that breast cancer had the best prognosis (median survival, 27.1 months), whereas gastrointestinal tumors had the worst (median survival, 2.66 months)^34^. According to Padalkar and Tow, the type of primary tumor was not statistically significant in the revised Tokuhashi scoring system based on five grades, while it was significant with the Tomita scoring system based on three grades^35^. Consequently, we categorized primary cancer types into three groups based on Tomita *et al*.^8^. However, we did not find that primary tumor influenced survival of patients with multiple spinal metastases. One possible explanation is that the presence of MSM itself is very aggressive, thus representing a more advanced stage of cancer compared to primary tumor types. Consequently, survival time of patients with MSM is equal to that of patients with different primary tumors.

Currently, only a few studies have focused on the prognostic value of the time of developing motor deficits before surgery in patients with MSCC^32^, ^36^, ^37^, ^38^, ^39^. Rades *et al*. showed that improved survival and function were associated with slower development of motor deficits^36^. Tabouret *et al*. found that a delay between the first symptom and surgery showed a tendency toward bad prognostic influence but failed to reach statistical significance^32^. Park reported that median survival for patients who developed neurologic deficit in less than 72 h was 2.28 times longer compared to patients who developed it ≥72 h (8.7 *vs* 3.1 months); the difference was statistically significant between groups^37^. Quraishi *et al*. reported that early surgical treatment (within 48 hours) did not produce a statistically significant correlation, but it did lead to significantly better neurological outcomes^38^, which is in line with a study conducted by Chaichana *et al*.^39^. In the present study, patients with solitary metastasis who developed motor deficit over a short period of time had a significantly better survival prognosis, while the same difference was not found in patients with multiple spinal metastases.

Serum albumin (SA), a symbol of nutritional status, is also used for predicting patient survival^33^, ^40^, ^41^, ^42^. Schoenfeld *et al*. revealed that preoperative SA levels ≥ 3.5 g/dL are strongly correlated with an increased chance of survival, especially for 30‐day survival. They also revealed that improved nutritional status may enhance postoperative survival of patients who undergo surgical intervention for MSCC^40^. Moreover, Ogihara *et al*. stated that the mean survival time in patients with SA < 3.0 g/dL is 3.1 months, and 7.4 months in patient with SA ≥ 3.0 g/dL, suggesting that SA level is a significant prognostic indicator for survival in patients with non‐small cell lung cancer^33^. Similarly, Ghori *et al*. identified SA as an independent predictor of 1‐year postoperative survival, and developed a scoring system that uses serum albumin as a prognostic factor^33^. Switlyk *et al*. found that survival rates at 6 and 12‐months were 63% and 46% in patients with SA ≥ 3.0 g/dL, and 15% and 15% in patients with SA < 3.0 g/dL, respectively; there was a significant difference between the two groups^42^. Our results, which were comparable with the aforementioned literature, revealed that patients with higher serum albumin levels had better prognosis.

## Limitations

This study has some limitations, which have to be pointed out. First, the design of the study was a retrospective review that included a small sample size and variable length of follow‐up period. Second, the study was based on a wide variety of primary tumors, while each primary tumor may show different biological behavior and different prognosis. It would be very useful to analyze the prognosis for individual tumor types in future studies, rather than analyzing all tumor types together. Third, the impact of the chemotherapy was not investigated in the present study because previous chemotherapy regimens varied among patients and these variations might have influenced survival. Fourth, it was difficult to decrease heterogeneity between the two groups due to diversity of MSCC characteristics. Nonetheless, we believe that the observed differences between the outcomes indicate a true difference resulting from treatments. Finally, the postoperative factors were excluded from the study because the inclusion of these data could lead to erroneous or confusing results. In addition, all authors agreed that analysis of postoperative factors provides no help in choice of surgical protocols because clinicians are not able to obtain postoperative data before treatment.

In summary, the number of spinal metastases had no statistically significant association with survival in patients with spinal metastases; more aggressive operations should be considered for patients with bone spinal and visceral metastasis. Nevertheless, it is necessary to consider prognostic differences in patients with multiple spinal metastases and solitary spinal metastasis.
